# Evaluating Various Ultrasound Criteria for Determining Carpal Tunnel Syndrome Severity

**DOI:** 10.1155/rrp/4936187

**Published:** 2025-08-19

**Authors:** Elaheh Mianehsaz, Hamidreza Talari, Marziyeh Naghavi Ravandi, Mohammad Hossein Tabatabaei, Mohammad Javad Azadchehr, Saeedeh Eshraqi, Mohammad Mahdi Heidari

**Affiliations:** ^1^Trauma Research Center, Kashan University of Medical Sciences, Kashan, Iran; ^2^Department of Radiology, Kashan University of Medical Sciences, Kashan, Iran; ^3^Department of Surgery, Kashan University of Medical Sciences, Kashan, Iran; ^4^Infectious Diseases Research Center, Kashan University of Medical Sciences, Kashan, Iran; ^5^Department of Pediatrics, School of Medicine, Iran University of Medical Sciences, Tehran, Iran

## Abstract

**Objective:** This study aimed at assessing the value of a variety of ultrasound criteria for grading carpal tunnel syndrome (CTS) severity.

**Methods:** Ultrasound evaluations were conducted on confirmed CTS patients by an experienced radiologist, blinded to NCS results. Cross-sectional area (CSA) at pronator quadratus muscle, carpal tunnel inlet and outlet, echogenicity, transverse motion during flexion, flattening ratio, and thickening of the flexor retinaculum were measured.

**Results:** Decreased echogenicity of the median nerve was notably observed as a distinguishing feature between mild and moderate cases. Decreased nerve movement was significantly more prevalent in severe CTS cases. No significant differences were found in the median nerve flattening ratio or flexor retinaculum thickness. Bowing at the inlet showed significant differences. CSA at the inlet and outlet indicated severe CTS with significant differences.

**Conclusion:** The findings highlight the importance of using multiple sonographic criteria for accurate diagnosis and treatment, although no significant differences were noted in the median nerve flattening ratio and flexor retinaculum thickness.

## 1. Introduction

Carpal tunnel syndrome (CTS) occurs when the median nerve, which runs from the forearm into the palm of the hand, is compressed at the wrist. This compression leads to symptoms such as pain, numbness, tingling, and weakness in the hand and fingers [1].

Electrodiagnostic methods, including electromyography (EMG) and nerve conduction velocity (NCV), are considered the gold standard for diagnosing CTS, with reported sensitivity ranging from 77% to 92% and specificity between 80% and 97%2. However, these techniques are relatively invasive and may cause discomfort for patients. EMG measures the electrical activity of muscles to identify damage due to nerve compression but lacks specificity for CTS. NCS effectively quantifies nerve compression but is more invasive than other modalities [2].

Ultrasound (US) has gained attention as a noninvasive alternative for diagnosing CTS. Unlike EMG and NCV, US examinations do not involve electrical stimulation, making them more comfortable for patients. Sonography provides real-time, dynamic images of the median nerve, carpal tunnel, and surrounding structures, enabling the identification of structural changes such as nerve enlargement, shape alterations (flattening or bulging), swelling, fluid collections, masses, and increased blood flow indicative of inflammation. Additionally, US can assess nerve movement, offering further diagnostic insights [3–7].

Several studies have highlighted the advantages of US over other imaging techniques. While MRI offers highly detailed images of wrist structures, it is more expensive and less accessible than US [8]. US's noninvasive nature, real-time imaging capability, and sensitivity to structural changes make it a promising modality for diagnosing CTS [9].

However, despite these benefits, there is a lack of consensus on which US criteria most accurately reflect the severity of nerve compression in CTS. This study aims to evaluate the value of different US criteria in determining the severity of CTS, thereby enhancing diagnostic accuracy and potentially informing treatment strategies.

## 2. Materials and Methods

### 2.1. Study Design and Participants

This cross-sectional study involved CTS patients, diagnosed via EMG-NCV as mild, moderate, or severe, at Shahid Beheshti Hospital, Kashan, Iran. Ethical approval (IR.KAUMS.MEDDNT.REC.1401.188) and informed consent were obtained. Participants diagnosed with conditions such as polyneuropathy, cervical radiculopathy, stroke, spinal cord injury, wrist fractures, dislocations, diabetes, hypothyroidism, or who had undergone prior CTS treatment were excluded from the study. Furthermore, EMG assessments were utilized to definitively rule out cervical radiculopathy and other potential neuropathies. US evaluations were performed on confirmed CTS patients diagnosed through EMG-NCV.

### 2.2. Electrodiagnostic Testing

CTS was diagnosed based on EMG and NCV criteria by a specialist in physical medicine and rehabilitation with over 10 years of experience. The diagnosis of CTS had to be supported by the findings of NCS, while EMG was performed to rule out other diagnoses such as cervical radiculopathy. The severity of the syndrome was determined as follows. Mild: Distal latency and sensory wave delay (sensory nerve action potential, SNAP) greater than 3.6 milliseconds (ms); distal SNAP latency difference between median and radial/ulnar nerves greater than 0.5 ms; median sensory distal latency difference (SNAP) between 14 cm (wrist) and 7 cm (palm) greater than 1.6 ms, with/without median SNAP amplitude less than 10 μV. Moderate: As above, plus motor wave (compound motor action potential, CMAP) distal latency greater than 4.2 ms, or distal latency difference between median and ulnar motor waves greater than 1 ms. Severe: As above, plus absence of SNAP wave or reduction in CMAP wave amplitude to less than 5 mV.

### 2.3. US Examination Procedures

The US examinations were performed using an A80WS UGEO Samsung model with a high-frequency linear probe (6.6–10 MHz). The US was performed by a radiologist with more than 5 years of experience in musculoskeletal sonography, who was blinded to the results of NCS in order to provide unbiased findings. Patients were seated with their arms extended, wrists resting on a flat, hard surface, forearms supinated, and fingers semiflexed. The US was conducted without applying pressure on the wrist. The transducer was placed transversely on the volar aspect of the wrist to capture images from the distal forearm to the carpal tunnel outlet. Measurements of the median nerve's cross-sectional area (CSA) were taken at several points: CSAp, distal third of forearm at the level of the proximal pronator quadratus muscle; CSAi, at the level of carpal tunnel inlet (level of the pisiform bone); and CSAo, carpal tunnel outlet (level of the hamate bone).

### 2.4. US Parameters Assessed

Ultrasound assessment focused on several key parameters. The CSA of the median nerve was measured at multiple anatomical locations to evaluate nerve size. Echogenicity was analyzed to characterize the internal echo texture of the nerve. The thickness of the flexor retinaculum was measured at the level of the carpal tunnel inlet. Bowing of the flexor retinaculum was quantified as the perpendicular distance from a reference line connecting the hook of the hamate to the tubercle of the trapezium, and from the pisiform and scaphoid bones to the flexor retinaculum. To assess nerve compression, the flattening ratio (FR) of the median nerve at the carpal tunnel inlet was calculated as the ratio of its major axis to minor axis. Median nerve mobility was assessed using dynamic transverse US imaging during active finger flexion and extension. The probe was held stationary in the transverse plane at the carpal tunnel inlet level. Normal mobility was defined as the visible displacement of the nerve deep to the flexor tendons, accompanied by changes in nerve morphology and vertical displacement relative to the overlying flexor retinaculum. Decreased mobility was recorded when transverse gliding, vertical motion, or shape change was absent or minimal. Mobility was qualitatively categorized as either normal or decreased.

### 2.5. Statistical Analysis

Data analysis was performed with the use of SPSS Version 23. Descriptive statistics consisted of mean and standard deviation for quantitative variables and number and percentage for qualitative variables. The analytical comparison for quantitative parameters was performed using the independent *T*-test and one-way ANOVA, whereas qualitative parameters were compared using the chi-square test. *p* < 0.05 was considered statistically significant.

## 3. Results

A total of 68 wrists were enrolled in this visit. Demographic data, as tabulated in Table 1, contain gender distribution, occupation, hand involvement, and symptom onset of the studied subjects.

NCS at the first visit reported involvement to be mild in 17 cases, that is, 25.0%, and moderate in 42 cases, that is, 61.7%, and in 9 cases or 13.3%, it was described as severe.

**Figure 1:**
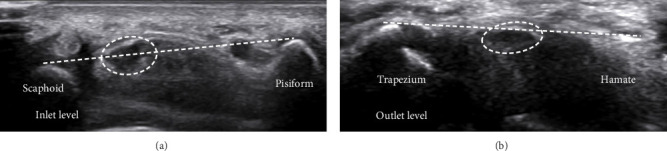
Ultrasound images of the median nerve at key anatomical landmarks. (a) The inlet level, showing the median nerve positioned between the scaphoid and pisiform bones. (b) The outlet level, demonstrating the median nerve near the trapezium and hamate bones.

The anatomical landmarks of the median nerve at the inlet and outlet levels of the carpal tunnel were carefully delineated using US imaging.  Figure 1 illustrates the median nerve's positioning relative to adjacent structures, including the Sca and Pis bones at the inlet level (Figure 1(a)) and the trapezium and hamate bones at the outlet level (Figure 1(b)).

Outlined below are the sonographic criteria assessed and their corresponding findings, with statistical comparisons summarized in Table 2.

### 3.1. Decreased Echogenicity of the Median Nerve

**Figure 2:**
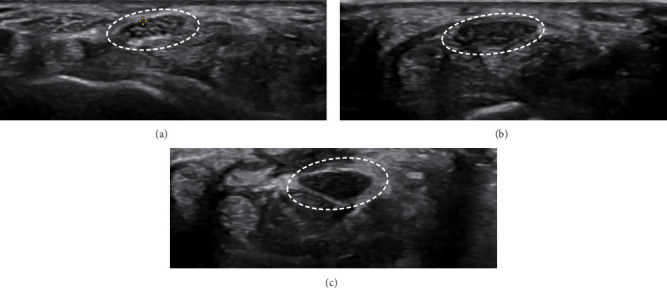
Ultrasound images of the median nerve in patients with varying severities of carpal tunnel syndrome (CTS). (a) Mild CTS, characterized by a distinct honeycomb-like pattern and preserved echogenicity. (b) Moderate CTS, showing a pronounced loss of the honeycomb pattern and further reduced echogenicity. (c) Severe CTS, exhibiting marked pattern loss and significant echogenicity reduction.

This was observed in 58.8% of mild cases, 88.1% of moderate cases, and 88.9% of severe cases. The overall statistical significance was marked with a *p* value of 0.028. In pairwise comparisons, the differences between mild and moderate cases were significant (*p* value: 0.011), while differences between mild and severe cases (*p* value: 0.114) and moderate and severe cases (*p* value: 0.947) were not as pronounced. A marked reduction in echogenicity was frequently observed in patients with severe CTS, suggesting a potential association between echogenicity changes and CTS severity (Figure 2). However, instances were noted where severe CTS cases did not exhibit significant reductions in echogenicity, reflecting variability in this feature among the study population.

### 3.2. Decreased Nerve Movement

**Figure 3:**
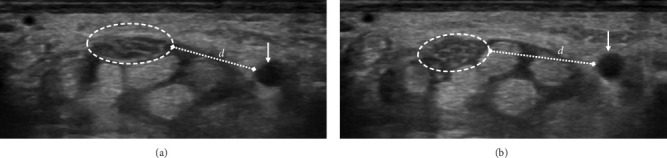
Transverse ultrasound imaging of the median nerve, highlighting its dynamic behavior and morphological changes during variations in finger positioning. (a) The median nerve (outlined by a dashed curved line) in the flexed position of the fingers, with its spatial relationship to the ulnar artery (arrow), along with observed alterations in nerve morphology. (b) The median nerve in the extended position of the fingers, illustrating its vertical translocation beneath the flexor tendon and concurrent changes in its structural profile. A decline in nerve mobility was defined as the absence of transverse-plane movement, lack of vertical displacement relative to the flexor tendon, or minimal morphological transformation during positional changes.

Dynamic US imaging was employed to assess the behavior and mobility of the median nerve during variations in finger positioning. Representative images are provided in Figure 3, highlighting the dynamic characteristics and morphological changes observed. The percentage of cases with decreased nerve movement was 29.4% in mild cases, 26.2% in moderate cases, and significantly higher at 88.9% in severe cases. The overall *p* value was 0.001, indicating statistical significance. In pairwise comparisons, significant differences were found between mild and severe cases (*p* value: 0.004) and between moderate and severe cases (*p* value: < 0.001), but not between mild and moderate cases (*p* value: 0.801).

### 3.3. Median Nerve FR

The FR was relatively consistent across the severity levels, with mild cases at 3.08 ± 1.08, moderate cases at 3.14 ± 0.89, and severe cases at 3.06 ± 0.38. This parameter did not show significant differences (overall *p* value: 0.953).

### 3.4. Thickness of the Flexor Retinaculum

The thickness measured in millimeters was 1.06 ± 0.11 in mild cases, 1.05 ± 0.08 in moderate cases, and 1.08 ± 0.04 in severe cases. The differences were not statistically significant (overall *p* value: 0.543).

### 3.5. Bowing at the Inlet of the Channel

The bowing at the inlet was observed to be 2.77 ± 1.10 mm in mild cases, 3.43 ± 0.92 mm in moderate cases, and 3.83 ± 0.91 mm in severe cases, with an overall *p* value of 0.018. Significant differences were noted between mild and severe cases (*p* value: 0.027), and a borderline significance was noted between mild and moderate cases (*p* value: 0.052).

### 3.6. Bowing at the Outlet of the Channel

The bowing at the outlet measured 1.50 ± 0.70 mm in mild cases, 1.62 ± 0.54 mm in moderate cases, and 1.82 ± 0.68 mm in severe cases. However, these differences were not statistically significant (overall *p* value: 0.452).

### 3.7. CSA of the Nerve at the Level of the Pronator Quadratus Muscle

The CSA was 6.70 ± 0.98 mm^2^ in mild cases, 6.74 ± 0.88 mm^2^ in moderate cases, and 7.78 ± 1.92 mm^2^ in severe cases. This criterion did not present significant differences (overall *p* value: 0.311).

### 3.8. CSA of the Nerve at the Inlet of the Canal

The CSA at the inlet showed significant differences, with measurements of 9.23 ± 1.85 mm^2^ in mild cases, 12.76 ± 2.26 mm^2^ in moderate cases, and 16.00 ± 3.00 mm^2^ in severe cases (overall *p* value: < 0.001). Pairwise comparisons revealed significant differences between mild and moderate cases (*p* value: < 0.001) and mild and severe cases (*p* value: < 0.001).

### 3.9. CSA of the Nerve at the Outlet of the Canal

The CSA at the outlet measured 6.35 ± 1.69 mm^2^ in mild cases, 9.56 ± 1.81 mm^2^ in moderate cases, and 9.33 ± 1.60 mm^2^ in severe cases. The overall *p* value was < 0.001, indicating significant differences between mild and moderate cases (*p* value: < 0.001) and mild and severe cases (*p* value: < 0.001).

### 3.10. Difference Between the CSA at the Inlet of the Canal and Pronator Quadratus Muscle

The difference in CSA was 2.53 ± 1.50 mm^2^ in mild cases, 6.02 ± 2.35 mm^2^ in moderate cases, and 8.22 ± 3.53 mm^2^ in severe cases. The overall *p* value was < 0.001, with significant differences in pairwise comparisons between mild and moderate cases (*p* value: < 0.001), mild and severe cases (*p* value: < 0.001), and moderate and severe cases (*p* value: 0.036).

**Figure 4:**
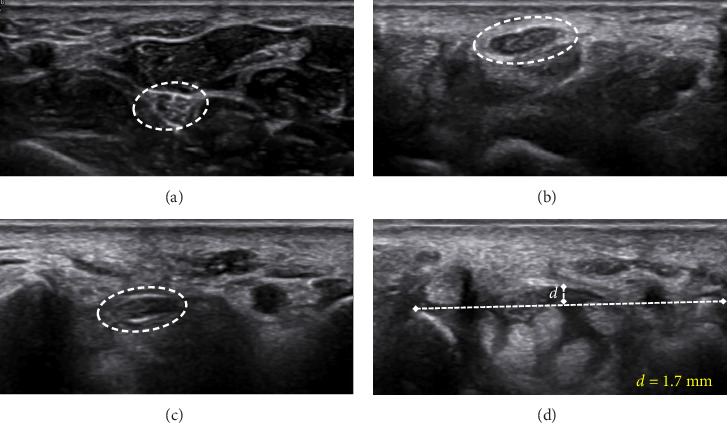
Transverse ultrasound image of the right median nerve in a female patient with mild CTS. (a) CSA at the pronator quadratus muscle (CSAp) is 0.06 cm^2^. (b) At the carpal tunnel inlet, CSA increased to 0.11 cm^2^. (c) The CSA at the carpal tunnel outlet is 0.09 cm^2^. (d) Bowing of the flexor retinaculum at the inlet is 1.7 mm.

**Figure 5:**
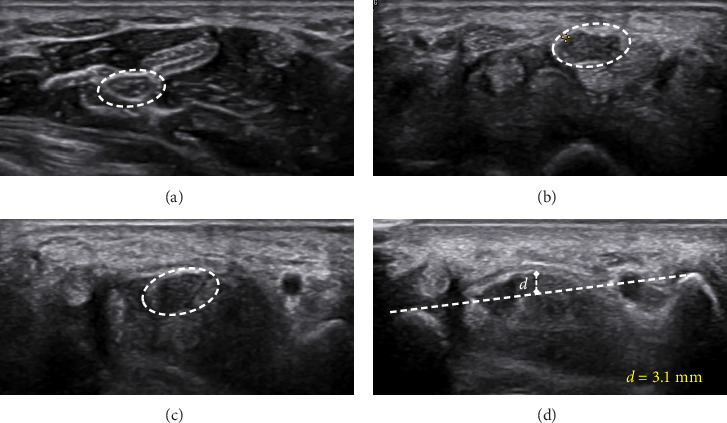
Transverse ultrasound of the right median nerve of a female with moderate CTS. (a) The CSAp is 0.06 cm^2^. (b) CSA at inlet of the carpal tunnel is 0.13 cm^2^. (c) CSA at outlet is 0.11 cm^2^. (d) The bowing of flexor retinaculum inlet is 3.1 mm.

**Figure 6:**
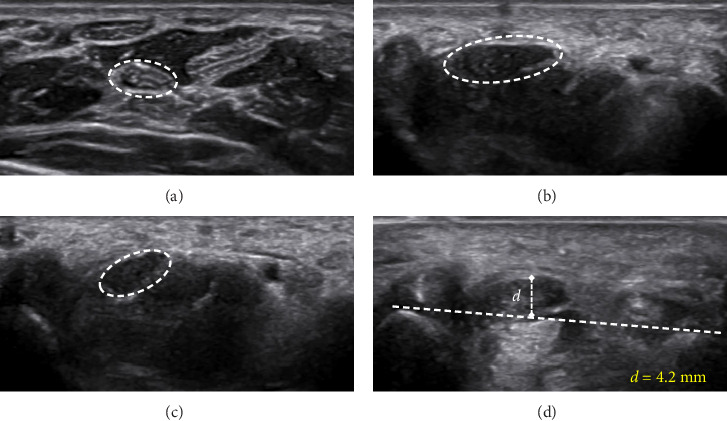
Transverse ultrasound image of the right median nerve in a male patient with severe CTS. (a) CSAp is 0.08 cm^2^. (b) CSA at the carpal tunnel inlet is 0.21 cm^2^. (c) CSA at the outlet is 0.14 cm^2^. (d) Bowing of the flexor retinaculum at the inlet is 4.2 mm.

Figures 4–6 present representative sonographic images that illustrate specific differences in the median nerve and surrounding structures among patients with various stages of CTS.

## 4. Discussion

The study population includes a significantly higher number of females (81.1%) compared to males (18.9%). This difference might indicate that the condition, possibly CTS, is more common among females due to various factors like biological, occupational, or lifestyle influences. A large portion of the participants are housekeepers (60.3%), which is an occupation known for tasks that involve repetitive hand movements, such as cleaning and cooking, potentially contributing to the development of such conditions. The next largest group comprises self-employed individuals (17.6%), who might also be engaged in jobs involving repetitive motions or heavy manual labor. Notably, most patients in our study presented with symptoms in both hands. For evaluation purposes, we selected the hand with more severe symptoms, which often turned out to be the dominant hand. This finding aligns with the hypothesis that repetitive use of the dominant hand could exacerbate CTS symptoms. On average, participants have been dealing with symptoms for about 13.11 months before seeking medical help, hinting at a delay in diagnosis or a tendency to endure symptoms until they become unmanageable.

During CTS diagnosis, the role of clinical examination is pivotal. However, more precise methods of investigation using NCS confirm the diagnosis, increase diagnostic accuracy for the syndrome, and establish its confirmation on a clinical basis. These methods, on the other hand, have a less acceptance rate because of being semi-invasive, costly, and with lesser accessibility. Recently, special attention has been directed toward noninvasive, cheaper, and more available methods like US, which has also been considered an efficient diagnostic tool. Despite the scant research regarding the accuracy and capability of US in the evaluation of the severity degree of CTS, the purpose of this study was to evaluate the diagnostic value of sonography compared with NCS regarding the assessment of CTS severity.

US offers dynamic imaging of the median nerve and the other structures through the carpal tunnel. Some sonographic features can help differentiate various stages of CTS, correlating with the underlying pathophysiology. In particular, both qualitative and quantitative US findings might be capable of determining the severity of this syndrome. The echogenicity of the median nerve and confirmation of decreased movement were relatively accurate to diagnose severe cases of the syndrome, 85.3%. Moreover, the CSA of the median nerve at the Pis level was effective in distinguishing between severe and nonsevere cases.

Sonographic imaging is an important modality for differentiating the stages in the development of CTS due to various changes that occur in the median nerve and surrounding structures [10]. In CTS, chronic compression and ischemia result in reduced echogenicity, with the median nerve becoming hypoechoic because of the increased water content and fibrosis, especially in advanced stages [11]. Results from this study showed a decrease of echogenicity in moderate CTS, compared to mild CTS. One limitation of this study was the low number of severe cases, as we had to exclude participants with a history of CTS therapy. This limitation negatively impacted our statistical analysis. For instance, we observed significant differences in the decrease of echogenicity between mild and moderate CTS, but not between mild and severe cases, due to the limited number of severe cases.

Other critical values include the CSA of the median nerve; an enlarged CSA is usually greater than 9-10 mm^2^, indicating nerve swelling and edema due to commonly accumulated extracellular fluid in moderate to severe CTS, accompanied by an inflammatory response [12]. Our study found that an enlarged CSA of the median nerve, typically greater than 11 mm^2^, is associated with moderate and greater than 14 mm^2^ is associated with severe CTS due to nerve swelling and edema. The CSA at the carpal tunnel outlet (CSAo) also showed increases, especially notable in moderate CTS. Additionally, the difference in CSA between the carpal tunnel inlet and the pronator quadratus muscle was larger in more severe cases, further supporting the role of nerve swelling in CTS progression. These findings highlight the diagnostic utility of CSA measurements in US imaging for accurately assessing CTS severity and guiding treatment plans.

The median nerve FR also gives a clear picture of the degree of compression; the higher the FR, the more severe the compression, where the nerve assumes a wider and thinner shape, characteristic of advanced stages. From our study, the median nerve FR did not show significant variation across different severities of CTS. The FR was 3.08 ± 1.08 in mild cases, 3.14 ± 0.89 in moderate cases, and 3.06 ± 0.38 in severe cases, with a *p* value of 0.953, indicating no statistically significant difference among the groups. This suggests that, in our cohort, the FR might not be as reliable a marker for distinguishing CTS severity as other parameters, such as the CSA of the median nerve. While the FR can theoretically indicate the degree of nerve compression, our findings imply that it may not consistently correlate with severity in a clinical setting. Further research with larger sample sizes could help clarify the role of the FR in assessing CTS severity.

Thickness of the flexor retinaculum may also indicate the severity of CTS, occurring due to fibrosis and increased pressure inside the carpal tunnel, higher in severe stages [13]. Our study found that the thickness of the flexor retinaculum did not vary significantly across different severities of CTS. Specifically, the mean thickness was 1.06 ± 0.11 mm in mild cases, 1.05 ± 0.08 mm in moderate cases, and 1.08 ± 0.04 mm in severe cases, with a *p* value of 0.543, indicating no significant difference. This suggests that, within our cohort, the flexor retinaculum thickness may not be a reliable marker for determining CTS severity. Further research is needed to clarify this relationship.

Limited nerve mobility due to adhesions and fibrosis from chronic compression also characterizes the progress to moderate and severe CTS, with limited gliding of the nerve inside the carpal tunnel [14]. Our study examined nerve movement status and found a significant reduction in mobility in severe CTS cases compared to mild and moderate cases (Table 2). This progression of reduced mobility aligns with the degree of compression, fibrosis, and adhesions typically seen in more advanced stages of CTS, which impede the gliding of the median nerve within the carpal tunnel. These findings underscore the importance of assessing nerve mobility in conjunction with other diagnostic parameters to accurately gauge the severity of CTS and inform appropriate treatment strategies. While transverse dynamic US is widely accepted and practical for assessing median nerve gliding, longitudinal displacement was not assessed in this study. Future studies could explore multiplanar dynamic assessments to provide a more comprehensive evaluation of nerve function.

Another characteristic feature of advanced CTS is bowing of the flexor retinaculum, where increased intracarpal pressure results in a convex curvature, accentuated in severe compression conditions [15, 16]. In our study, the degree of bowing of the flexor retinaculum was significantly associated with the severity of CTS. The data showed that the mean bowing at the inlet of the carpal tunnel was 2.77 ± 1.10 mm in mild cases, 3.43 ± 0.92 mm in moderate cases, and 3.83 ± 0.91 mm in severe cases, with a *p* value of 0.018. This indicates that the bowing of the flexor retinaculum increases with CTS severity, likely due to higher intracarpal pressure and resultant structural changes in more advanced stages. The pairwise comparisons also showed significant differences between mild and severe cases (*p*=0.027), highlighting that this sonographic feature can serve as a reliable indicator of severe compression conditions. These findings underscore the importance of assessing the bowing of the flexor retinaculum as part of a comprehensive sonographic evaluation for accurately determining CTS severity.

These sonographic features reflect the basic theory and pathophysiology of CTS, providing a comprehensive view of disease progression. A combination of these sonographic features allows the clinician to better grade the severity of CTS [17].

The field of US in detecting the severity of CTS has become a hot topic in recent research [18, 19]. For instance, El-Maghraby et al. explored the combined diagnostic utility of B-mode US and shear wave elastography (SWE) for assessing CTS severity, showing that these methods could serve as effective alternatives to electrodiagnostic tests [20]. Similarly, Prakash et al. demonstrated that high-frequency grayscale ultrasonography and sonoelastography could accurately grade CTS severity in comparison to NCSs [21]. Azami et al. compared US and NCV in diagnosing CTS. They found that US parameters, such as the CSA of the median nerve, were effective in diagnosing of CTS [22]. Aseem et al. evaluated the role of US in conjunction with NCS in diagnosing and evaluating the CTS. They found that US, particularly the CSA at the tunnel inlet, had good diagnostic value [23]. Ooi et al. investigated the relationship between US and electrodiagnostic findings in CTS. They found significant correlations between sonographic parameters, such as CSA and FR, and the severity of CTS based on electrodiagnostic studies [24]. Moreover, Lee et al. investigated the predictive value of grayscale and power Doppler US findings in assessing cubital tunnel syndrome severity, showing that these methods could offer valuable insights into the extent of the disease [25]. Although our results were aligned with those of other studies in most criteria, our findings showing decreased echogenicity of the median nerve were not emphasized in other studies.

## 5. Conclusion

This study has identified the diagnostic value of US in assessing the severity of CTS. The median nerve CSA, as a sonographic criterion, increasingly and significantly rose with the deterioration of CTS. Higher severities of CTS had increased bowing of the flexor retinaculum due to increased intracarpal pressure. Reduced nerve mobility also appeared in the great majority of subjects with moderate and severe stages. There were no statistical differences regarding the FR of the median nerve and the thickness of the flexor retinaculum. Overall, these results emphasize the utility of a combined approach using multiple sonographic criteria in the accurate diagnosis of the severity of CTS and proper treatment planning.

## Figures and Tables

**Table 1:** Demographic and clinical characteristics.

Variables	All patients (*n* = 68)
Mean age (years)	47.43 ± 9.56

Mean body mass index (BMI, kg/m^2^)	28.87 ± 4.91

Gender	
Male	13 (18.9%)
Female	55 (81.1%)

Occupation	
Housekeeper	41 (60.4%)
Office worker	9 (13.2%)
Manual laborer	6 (8.8%)
Self-employed	12 (17.6%)

Dominant hand	
Right	64 (94.1%)
Left	4 (5.9%)

Mean duration of onset of symptoms (months)	13.11 ± 11.24

**Table 2:** The association between the severity of carpal tunnel syndrome based on NCS findings and sonography findings.

Index	Severity of carpal tunnel syndrome			Overall *p* value	*p* value (pairwise)
	Mild (*n* = 17)	Moderate (*n* = 42)	Severe (*n* = 9)		
Decreased echogenicity of the median nerve	10 (58.8%)	37 (88.1%)	8 (88.9%)	0.028	Mild-moderate: *p* < 0.05
					Mild-severe: *p*=0.114
					Moderate-severe: *p*=0.947

Decreased nerve movement	5 (29.4%)	11 (26.2%)	8 (88.9%)	0.001	Mild-moderate: *p*=0.801
					Mild-severe: *p* < 0.01
					Moderate-severe: *p* < 0.001

Median nerve flattening ratio	3.08 ± 1.08	3.14 ± 0.89	3.06 ± 0.38	0.953	—

The thickness of the flexor retinaculum (mm)	1.06 ± 0.11	1.05 ± 0.08	1.08 ± 0.04	0.543	—

Bowing at the inlet of the channel (mm)	2.77 ± 1.10	3.43 ± 0.92	3.83 ± 0.91	0.018	Mild-moderate: *p*=0.052
					Mild-severe: *p* < 0.05
					Moderate-severe: *p*=0.508

Bowing at the outlet of the channel (mm)	1.50 ± 0.70	1.62 ± 0.54	1.82 ± 0.68	0.452	—

CSA of the nerve at the level of the pronator quadratus muscle (mm^2^)	6.70 ± 0.98	6.74 ± 0.88	7.78 ± 1.92	0.311	—

CSA of the nerve at inlet of canal (mm^2^)	9.23 ± 1.85	12.76 ± 2.26	16.00 ± 3.00	< 0.001	Mild-moderate: *p* < 0.001
					Mild-severe: *p* < 0.001
					Moderate-severe: *p* < 0.001

CSA of the nerve at outlet of canal (mm^2^)	6.35 ± 1.69	9.56 ± 1.81	9.33 ± 1.60	< 0.001	Mild-moderate: *p* < 0.001
					Mild-severe: *p* < 0.001
					Moderate-severe: *p*=0.944

The difference between the CSA of the nerve at the inlet of canal and pronator quadratus muscle (mm^2^)	2.53 ± 1.50	6.02 ± 2.35	8.22 ± 3.53	< 0.001	Mild-moderate: *p* < 0.001
					Mild-severe: *p* < 0.001
					Moderate-severe: *p* < 0.05

## Data Availability

The data that support the findings of this study are available on request from the corresponding author. The data are not publicly available due to privacy or ethical restrictions.
